# Soluble ephrin a1 is necessary for the growth of HeLa and SK-BR3 cells

**DOI:** 10.1186/1475-2867-10-41

**Published:** 2010-10-27

**Authors:** Spencer Alford, Adam Watson-Hurthig, Nadia Scott, Amanda Carette, Heather Lorimer, Jessa Bazowski, Perry L Howard

**Affiliations:** 1 Department of Chemistry, University of Alberta, 11227 Saskatchewan Drive, Edmonton, Alberta, T6G 2G2, Canada; 2 Department of Biochemistry and Microbiology, University of Victoria, P.O. Box 3055 Station CSC Victoria, British Columbia, V8W 3P6, Canada; 3 Department of Biology, University of Victoria P.O. Box 3020 Station CSC Victoria, British Columbia, V8W 3N5, Canada; 4 Centre for Biomedical Research, University of Victoria, P.O. Box 3020 Station CSC Victoria, British Columbia, V8W 3N5, Canada

## Abstract

**Background:**

Ephrin A1 (EFNA1) is a member of the A-type ephrin family of cell surface proteins that function as ligands for the A-type Eph receptor tyrosine kinase family. In malignancy, the precise role of EFNA1 and its preferred receptor, EPHA2, is controversial. Several studies have found that EFNA1 may suppress EPHA2-mediated oncogenesis, or enhance it, depending on cell type and context. However, little is known about the conditions that influence whether EFNA1 promotes or suppresses tumorigenicity. EFNA1 exists in a soluble form as well as a glycophosphatidylinositol (GPI) membrane attached form. We investigated whether the contradictory roles of EFNA1 in malignancy might in part be related to the existence of both soluble and membrane attached forms of EFNA1 and potential differences in the manner in which they interact with EPHA2.

**Results:**

Using a RNAi strategy to reduce the expression of endogenous EFNA1 and EPHA2, we found that both EFNA1 and EPHA2 are required for growth of HeLa and SK-BR3 cells. The growth defects could be rescued by conditioned media from cells overexpressing soluble EFNA1. Interestingly, we found that overexpression of the membrane attached form of EFNA1 suppresses growth of HeLa cells in 3D but not 2D. Knockdown of endogenous EFNA1, or overexpression of full-length EFNA1, resulted in relocalization of EPHA2 from the cell surface to sites of cell-cell contact. Overexpression of soluble EFNA1 however resulted in more EPHA2 distributed on the cell surface, away from cell-cell contacts, and promoted the growth of HeLa cells.

**Conclusions:**

We conclude that soluble EFNA1 is necessary for the transformation of HeLa and SK-BR3 cells and participates in the relocalization of EPHA2 away from sites of cell-cell contact during transformation.

## Background

The Eph receptors are the largest family of receptor tyrosine kinases. They are activated by protein ligands, known as ephrins, which are attached to the cell membrane by either a membrane-spanning protein domain (B-type) or by a glycosylphosphatidylinositol (GPI) anchor (A-type). The receptors are also divided into A and B classes according to the type of ephrin they bind and their sequence similarity. Typically, the Eph A receptors bind to A-type ephrins, and Eph B receptors bind to B-type ephrins. However, binding between classes does occur with certain family members [1,2]. The functions regulated by Eph receptors and their ephrin ligands are diverse and cell-type dependent. They control a large number of physiological and developmental processes, and have also been implicated in both the suppression and advancement of cancer (reviewed in [3]).

Perhaps the best characterized, in terms of its pro- and anti-oncogenic roles, is EPHA2. EPHA2 confers tumorigenic and metastatic potential to non-transformed breast and skin epithelial cells, as well as mouse fibroblasts, and is overexpressed in tumor parenchyma of several cancers, including breast, bladder, prostate, colon, eosophageal, ovarian, cervical, stomach, and melanoma [4-12]. This ability to transform fibroblasts and some epithelial cell types, as well as its high expression levels in several different types of cancer, suggests that EPHA2 may have a direct role in oncogenesis. Independent reports support this hypothesis. For example, analysis of EPHA2 knockout mice revealed that EPHA2 enhances ErbB2-mediated tumorigenesis in MMTV-Neu mammary tumor mouse models[13]. As well, EPHA2 knockout mice are deficient in their ability to support the invasion and metastasis of implanted tumors, likely through a defect in angiogenesis[14]. In contrast to this pro-oncogenic role for EPHA2, EPHA2 knockout mice are more susceptible to chemically-induced skin cancer, which indicates that in some circumstances EPHA2 can suppress tumorigenesis [15]. However, little is known about what factors determine whether EPHA2 augments or suppresses cancer progression.

One feature that appears to distinguish oncogenic EPHA2 from the tumor suppressive form is its cellular localization. In non-transformed cells, EPHA2 is localized primarily to cell-cell junctions. Conversely, in transformed cells, EPHA2 is distributed on the cell surface and is localized to membrane ruffles [4,16-19]. The localization of EPHA2 and stability of adherens junctions are intimately linked. Ephrin stimulation of EPHA2 activity in normal epithelial cells enhances cell-cell adhesions by suppressing Arf6 GTPase and loss of E-cadherin results in EPHA2 mislocalization[16,19]. Conversely overexpression of EPHA2 increases the turnover of E-cadherin cell adhesions in a RhoA dependent manner and leads to EPHA2 mislocalization [17]. Thus the localization of EPHA2 correlates functionally with its roles in growth suppression and oncogenesis.

One of the contextual factors affecting EPHA2 transformation is likely the expression of its preferred ligand, EFNA1. EFNA1 is often co-expressed in tumors along with EPHA2[7,20-25] EFNA1 can both inhibit and stimulate oncogenesis, depending on the cellular context. In some cell types, such as glioblastoma multiforme cells, EFNA1 expression downregulates EPHA2 and suppresses EPHA2-mediated oncogenesis[26,27]. Indeed, stimulation of certain EPHA2 overexpressing cancer cell lines with recombinant EFNA1-Fc fusion proteins has been shown to suppress oncogenesis by causing receptor internalization [4,27-29] and in normal epithelial cells EFNA1 functions at cell-adhesions to stabilize E-cadherin adhesion complexes [19]. However, the suppressive effects of EFNA1 are not ubiquitously observed and several studies have supported a pro-oncogenic role for EFNA1. There are many cancers and cancer cell lines that overexpress both EPHA2 and EFNA1, including bladder and ovarian cancer, which indicates that EFNA1 expression does not always lead to EPHA2 downregulation [24,25]. As an example, in HT29 colorectal cancer cells, Potla et al. (2002) showed that endogenous EFNA1 is required for the growth in semi-solid media of these EPHA2 positive cells [23]. A positive role in promoting cancer has been confirmed experimentally in transgenic mice overexpressing EFNA1 in the intestinal mucosa, where EFNA1 enhances malignant progression in Apc^min/+^mice[30]. Therefore, the ability of EFNA1 to suppress or contribute to EPHA2-mediated oncogenesis appears to be contextual and cell-type dependent. However, the exact physiological conditions that elicit tumor suppression or oncogenesis remain elusive.

In addition to the GPI-linked membrane bound form of EFNA1, EFNA1 is also shed from the cell surface through the action of lipases and metalloproteases [7,21,31]. In fact, soluble EFNA1 is present in conditioned media from numerous cancer cell lines and was originally described as a soluble angiogenic factor induced by TNFα [21,32,33]. Until recently, this soluble pool of EFNA1 was thought to be inactive. This assumption was based on early studies that showed that ephrins require membrane attachment and higher-order clustering to stimulate Eph receptor activity [34]. Recently, however, it has been shown that soluble monomeric EFNA1 can activate EPHA2 activity [31]. This study supports the early work of Bartley et al.(1994) which showed that EFNA1 purified from conditioned media could induce EPHA2 phosphorylation and was important for TNFα induced angiogenesis [7,21,33]. The existence of a soluble isoform, in addition to a membrane attached isoform, implies that EFNA1 can also function at a distance away from immediate cell-cell contacts. We reasoned that the reported contradictory effects of EFNA1 in malignancy might in part be related to the existence of both soluble and membrane attached forms of EFNA1 and potential differences in the manner in which they interact with EPHA2.

To address this issue, we used RNAi to reduce EFNA1 expression in HeLa and SK-BR3 cells, which express both EPHA2 and EFNA1 endogenously, and also produce a soluble isoform of EFNA1. Here we show that soluble EFNA1 is necessary for the growth of these cells. In addition, we show that soluble EFNA1 contributes to the EPHA2 relocalization from sites of cell-cell contact to the cell surface, which occurs during epithelial cell transformation.

## Results

**Figure 1:**
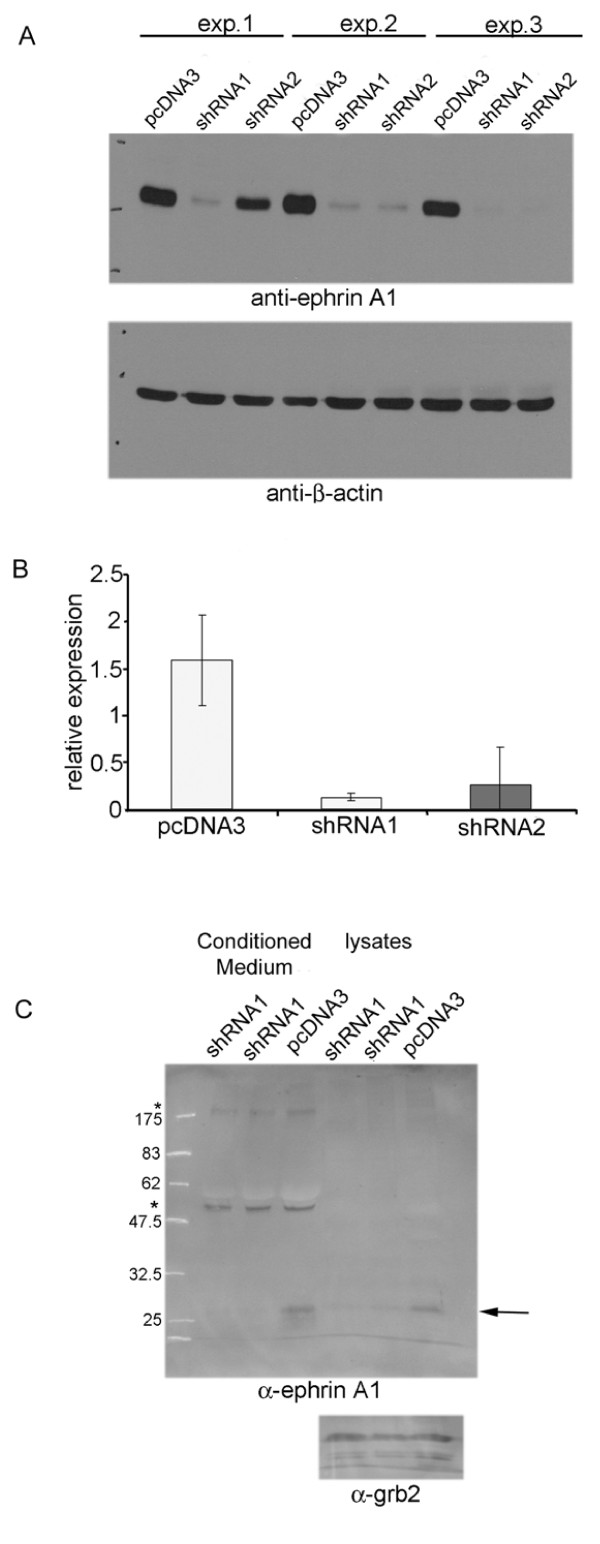
**Knockdown of exogenous and endogenous EFNA1**. For exogenous knockdown of EFNA1, HeLa cells were transiently transfected with EFNA1 cDNA and with either empty vector control, EFNA1 shRNA#1, or EFNA1 shRNA#2 plasmids. A) Western blot of 3 independent experiments showing the typical level of knockdown of exogenous EFNA1 (top). Blot was stripped and reprobed with an anti-β-actin antibody to show equal loading between lanes. B) Quantification of knockdown showing the average expression of EFNA1 relative to the amount of β-actin +/- the standard deviation. C) Endogenous EFNA1 knockdown. HeLa cells were transiently transfected with either empty vector control, or EFNA1 shRNAi#1. Shown are duplicate transfections. Western blotting using an anti-EFNA1 antibody showed a knockdown of endogenous EFNA1 in both conditioned medium and lysates (arrow). Below is a western blot using anti-Grb2 which was used to confirm equal loading of lysate lanes. * shows two cross-reacting bands which were used to confirm equal loading of conditioned medium lanes.

To examine the role of endogenous soluble and membrane bound EFNA1 in the context of EPHA2 oncogenesis, we chose HeLa cells, an aggressive cervical cancer cell line that expresses both EPHA2 and EFNA1 (Figure 1c, 2c). To determine the function of endogenous EFNA1 expression, we first developed shRNA directed against EFNA1. Hairpin oligos targeting two different sequences of human EFNA1 were generated and cloned into a mammalian expression vector. Expression of the shRNAs reduced the expression of an exogenously expressed EFNA1, confirming the ability of the shRNA to target EFNA1 (Figure 1a,b). To determine whether the endogenous EFNA1 was attached to the cell surface or released, conditioned media and lysates were prepared from cultured cells. Conditioned media was collected after 48 hours of incubation, concentrated 20-fold (to approximately same volume as lysates), and analyzed by Western blot. A 25 kDa band corresponding to EFNA1 was detected in the cellular lysates, as well as in the conditioned media from HeLa cells (Figure 1c). To confirm that this band corresponded to EFNA1, we knocked down EFNA1 expression in HeLa cells using shRNA. Transfection of shRNA directed against EFNA1 decreased the expression of EFNA1 in both the lysates and conditioned medium as expected (Figure 1c). In the conditioned media from HeLa cells, we also detected two high molecular weight bands at approximately 50 kDa and 200 kDa (Figure 1c). Soluble EFNA1 has been reported to be a substrate for tissue transglutaminase, which covalently oligomerizes the soluble A-type ephrins [35]. However, we did not detect a decrease in the levels of these proteins following shRNA transfection, suggesting that they represent non-specific antibody binding rather than oligomerized forms of EFNA1. Our finding of EFNA1 in the conditioned media of HeLa cells indicates that, like many cancer cells, HeLa cells express EFNA1 in addition to EPHA2, and that some of the EFNA1 is released from the cell surface of these cells. The existence of soluble EFNA1 in HeLa cells raises the possibility that it can affect EPHA2 signaling.

**Figure 2:**
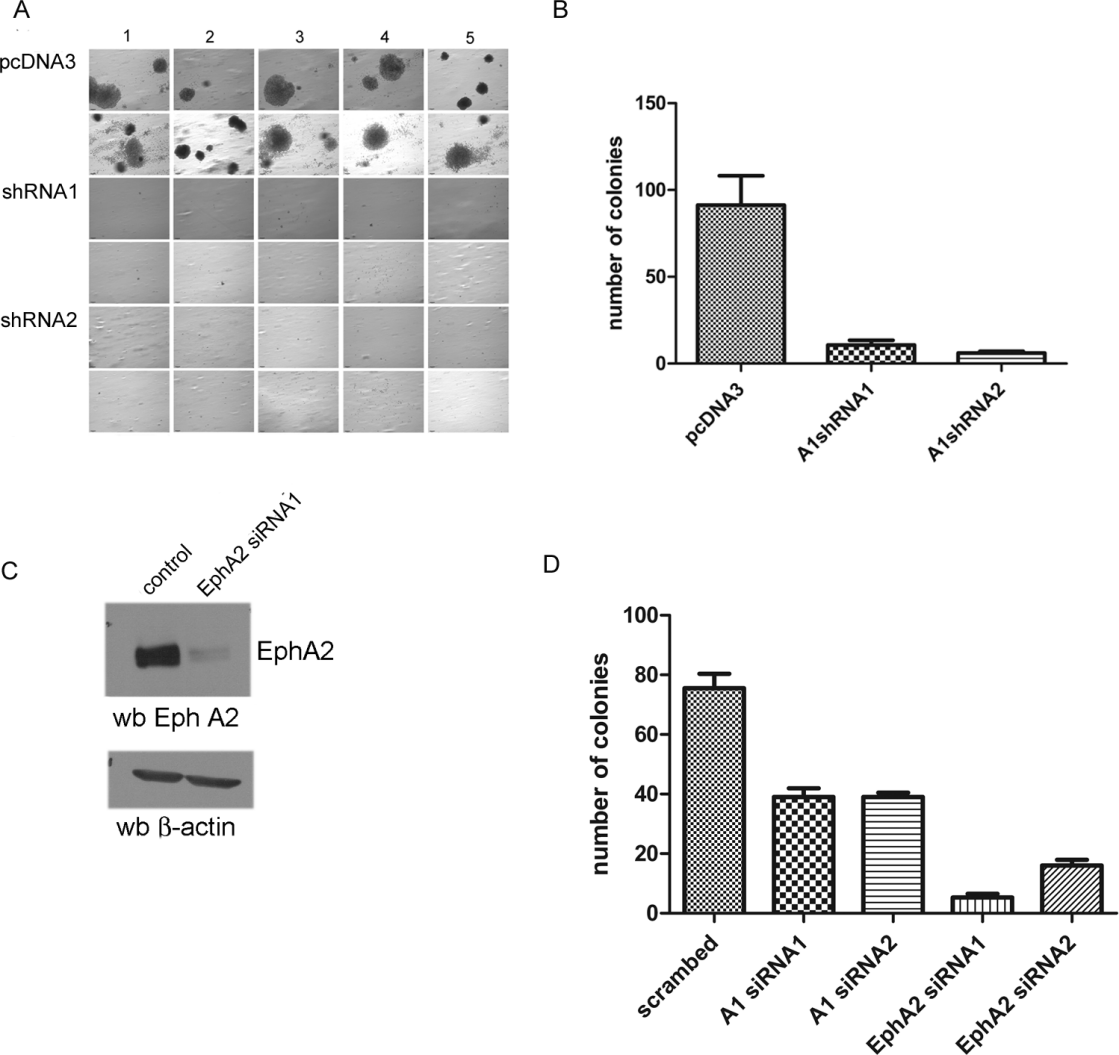
**Knockdown of EFNA1 and EPHA2 suppresses growth in semi-solid medium**. A) HeLa cells were transfected with pcDNA3 empty vector control, EFNA1 shRNA1, EFNA1 shRNA2. Transfected cells were plated in 0.5% agarose and allowed to grow for two weeks. Shown are ten representative fields for each transfection. B) Quantification of growth in semi-solid medium. Results represent the average number of colonies +/- standard deviation from three independent experiments each plated in quadruplicate. C) Western blot of knockdown of endogenous EPHA2 showing typical knockdown of using siRNA directed against EPHA2 (Top). Below is the same blot reprobed with anti-β-actin, which was used to confirm equal loading between lanes. D) Quantification of growth in semi-solid medium as above. In this case, HeLa cells were transfected with siRNAs against EFNA1 or EPHA2.

Although it is known that EPHA2 can transform several cell types, the role of EFNA1 in this process is not known. The inherent co-expression of both EPHA2 and EFNA1 in HeLa cells gave us the opportunity to address the role of endogenous EFNA1 in EPHA2-mediated oncogenic signaling. We characterized the effect of EFNA1 knockdown on the growth of HeLa cells in semi-solid media. Growth in semi-solid media is a characteristic trait of most cancer cells, and is widely used as an *in vitro *measure of tumorigenicity [36]. Knockdown of EFNA1 reduced the ability of HeLa cells to grow in semi-solid medium (Figure 2a, b). This result indicates that EFNA1 is required for the anchorage independent growth of HeLa cells. To knockdown EPHA2, commercially available short interfering RNA (siRNA) duplexes were used. For comparison purposes, siRNA duplexes targeting the identical sequences as the EFNA1 shRNA were also generated. Similar to the effects observed for EFNA1 knockdown, siRNA-mediated knockdown of EPHA2 reduced the level of expression of EPHA2 in HeLa cells (Figure 2c) and inhibited the ability of the cells to grow in semi-solid medium (Figure 2d). These results indicate that, like HT29 colon carcinoma cells, HeLa cells require both EFNA1 and EPHA2 for anchorage independent growth [23].

**Figure 3:**
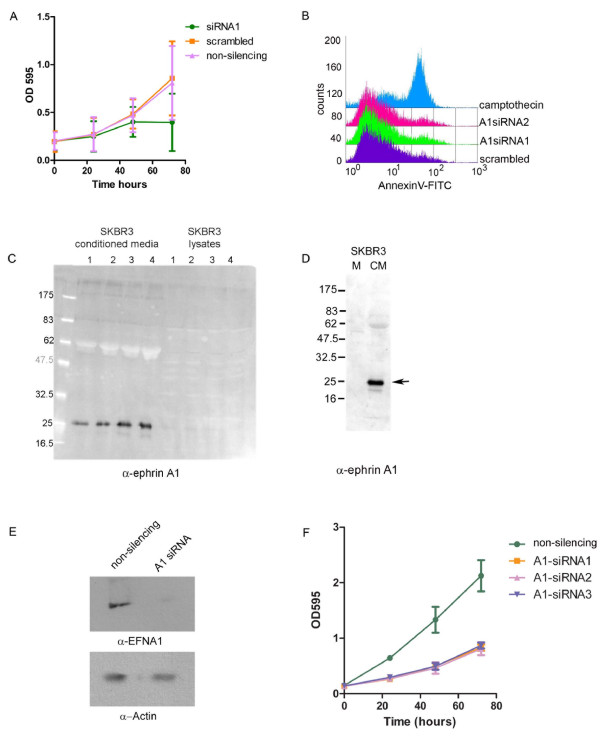
**EFNA1 is required for growth of HeLa and SK-BR3 cells**. A) HeLa cells were plated at low density and transfected with either scrambled siRNA, a universal non-silencing siRNA, or EFNA1 siRNA. Growth was monitored over a 72 hour period using crystal violet staining. B) Knockdown of EFNA1 with siRNA1 or siRNA2 did not affect apoptosis levels in HeLa cells and were comparable to scrambled siRNA treated cells. Shown is a representative histogram of AnnexinV staining on transfected cells 24 hours after transfection. Camptothecin treatment was used as a positive control for apoptosis. C) Western blot of endogenous EFNA1 expression in SK-BR3 cell lysates and conditioned media from these cells. D) Western blot of EFNA1 showing band detected in conditioned media is not present in media alone. E) Western blot of SK-BR3 cells transfected with EFNA1 siRNA. Below is the same blot reprobed with anti-β-actin, which was used to confirm equal loading between lanes. F) SK-BR3 cells were plated at low density and transfected with either, non-silencing siRNA, or 3 different EFNA1 siRNA. Growth was monitored over a 72 hour period using crystal violet staining.

We next examined the effects of EFNA1 knockdown on growth in two dimensions (2D). We observed that a decrease in EFNA1 expression reduced the growth of HeLa cells in 2D (Figure 3a). Unlike the results of Potla et al. (2002) using HT29 cells, the effect on growth did not appear to be density dependent [23]. We did not detect an increase in apoptosis, indicating that EFNA1 is required for the growth of HeLa cells but not for survival (Figure 3b). To test whether this requirement for EFNA1 is restricted to HeLa cells, we also knocked down EFNA1 in SK-BR3 cells. These cells are an invasive breast epithelial cancer cell line that express soluble EFNA1 at high levels (Figure 3c,d)[21]. Similar to HeLa cells, knockdown of EFNA1 in SK-BR3 cells caused a decrease in cell proliferation (Figure 3e,f). Since most of the EFNA1 in these cells is found in the media, this result suggests that soluble EFNA1 is required to promote the growth of these cancer cells.

**Figure 4:**
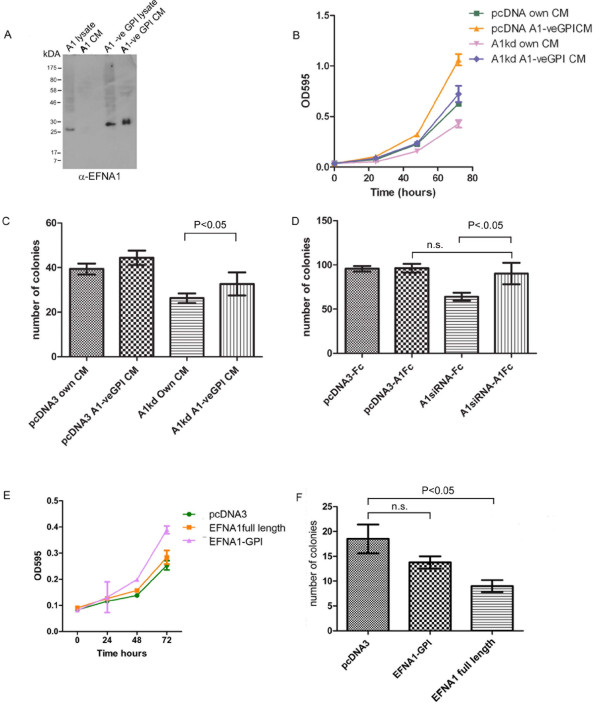
**Transfection of HeLa cells with full length EFNA1 (A1) or a truncated EFNA1 lacking the C-terminal signal for GPI anchor attachment (A1-ve GPI)**. Shown is an anti-EFNA1 western blot of lysates and conditioned media. Truncated EFNA1 accumulates in the conditioned medium whereas the full-length EFNA1 does not. B) Soluble EFNA1 in conditioned media rescues the growth of EFNA1 knockdown cells. HeLa cells stably transfected with either vector control or shRNA against EFNA1 were grown in conditioned media from either vector control or knockdown cells (own CM) or conditioned media from cells in which truncated soluble EFNA1 is overexpressed (A1-veGPI CM). C) Soluble EFNA1 in conditioned media partially rescues the growth of EFNA1 knockdown cells in semi-solid media. HeLa cells stably transfected with either vector control or shRNA against EFNA1 were grown in semi solid media that was supplemented with either conditioned media from either vector control or knockdown cells (own CM) or conditioned media from cells in which truncated EFNA1 is overexpressed (A1-veGPI CM). D) EFNA1-Fc rescues the growth of EFNA1 knockdown cells in semi-solid media. HeLa cells stably transfected with either vector control or shRNA against EFNA1 were grown in semi media that was supplemented with either EFNA1-Fc (A1Fc) or Fc alone. E) Overexpression of soluble EFNA1 (EFNA1-GPI) but not full length EFNA1 promotes the growth of HeLa cells. HeLa cells were stably transfected with pcDNA3, EFNA1 full length, or truncated EFNA1-GPI. Pools of stable clones were then counted, seeded at low density, and growth was monitored by crystal violet staining. F) Overexpression of EFNA1 inhibits the growth of HeLa cells in semi-solid media. The stable pools of HeLa cells described above were plated at the same density in semi-solid media and the number of colonies determined after two weeks of growth.

To test the hypothesis that soluble EFNA1 is necessary for growth of HeLa cells, we determined if soluble EFNA1 was sufficient to rescue the growth of HeLa cells in 2D. Conditioned media from HeLa cells, EFNA1 knockdown cells, or HeLa cells stably transfected with a truncated version of EFNA1 (lacking the C-terminal sequence necessary for GPI anchor attachment) were collected and used to treat either vector control HeLa cells, or the HeLa cells in which EFNA1 expression has been stably knocked down. As shown in Figure 4a, expression of the truncated EFNA1 increases the amount of soluble EFNA1 in the media, and therefore provides an enriched source of soluble EFNA1. Conditioned media from cells overexpressing soluble EFNA1 rescued the growth of EFNA1 knockdown cells as well as enhanced the growth of the vector control cells (Figure 4b). Next we tested whether soluble EFNA1 collected from the overexpressing cells was sufficient to rescue growth in semi-solid media of EFNA1 knockdown cells. Treatment of knockdown cells with conditioned media from soluble EFNA1 overexpressing cells partially rescued anchorage independent growth. We suspected that the inability of the soluble EFNA1 to completely rescue the growth in semi-solid agarose was due to limited amounts of EFNA1 in the conditioned media. In our rescue of the 2D growth of EFNA1 knockdown lines, we observed that growth was improved when the conditioned media was changed frequently. This was not feasible with the prolonged culture time of the 3D experiments. In support of this, treating cells with EFNA1-Fc fusion protein (1 μg/ml) rescued the growth in semi-solid agarose of EFNA1 knockdown cells (Figure 4d). These results show that soluble EFNA1 is necessary for the growth of HeLa cells in both 2D and 3D.

To explore whether there are differences in the signaling properties of soluble and membrane bound EFNA1, we overexpressed full-length EFNA1, or soluble EFNA1, in HeLa cells and compared the ability of the transfected cells to grow in 2D. This experiment was based on the fact that the expression of the full length construct did not significantly increase the amount of soluble EFNA1 (Figure 4A). Overproduction of soluble EFNA1 promoted the growth of HeLa cells in 2D whereas overexpression of full length EFNA1 had no effect, which confirms that soluble EFNA1 has pro-growth effects on these cells (Figure 4e). Interestingly, expression of full-length EFNA1 suppressed the ability of HeLa cells to grow in semi-solid medium (Figure 4f). Cells expressing soluble EFNA1 appeared to be intermediate between the control and full-length EFNA1. This suggests overproduction of soluble EFNA1 may be slightly inhibitory, although this effect was not statistically significant (Figure 4f). These results indicate that overproduction of EFNA1 inhibits anchorage independent growth.

**Figure 5:**
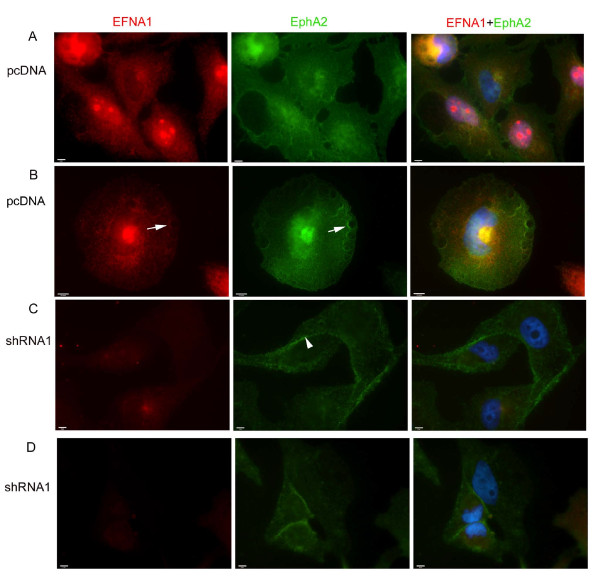
**Localization of EFNA1 and EPHA2 in knockdown and control cells. HeLa cells were transfected with empty vector (pcDNA), shRNA1 against EFNA1 (shRNA) and the localization of EPHA2 and EFNA1 was determined**. Shown are representative images. In control cells (pcDNA A and B), EFNA1 and EPHA2 colocalize on the dorsal surface of the cell and to membrane ruffles (arrows) with very little expression at cell-cell contacts. In EFNA1 knockdown cells (shRNA A1 C and D), EPHA2 was localized primarily to cell-cell contacts (arrowhead) at the peripheral membrane. Bar represents 5 μm.

**Figure 6:**
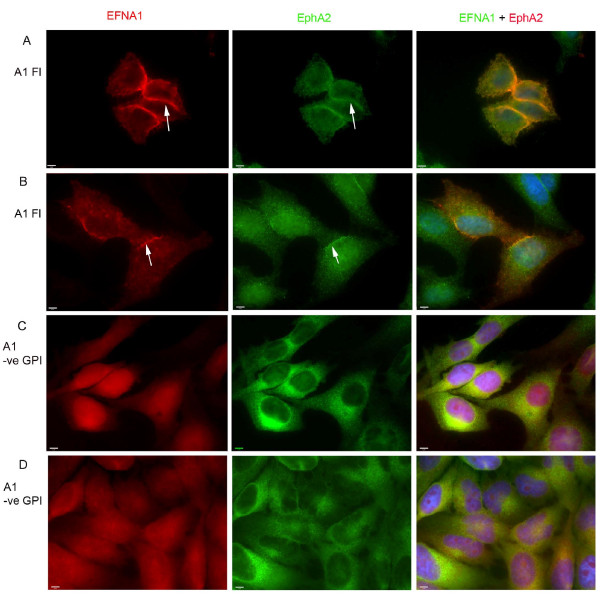
**Localization of EFNA1 and EPHA2 in cells overexpressing EFNA1**. In cells transfected with full length EFNA1 (A1 FL A and B), EPHA2 strongly colocalized with EFNA1 at cell-cell contacts (arrows) and exhibited less cell surface staining. In cells transfected with truncated soluble EFNA1 (A1-veGPI C and D), EPHA2 and EFNA1 colocalized to the dorsal surface of cells and were not enriched at cell-cell junctions.

In normal epithelial cells, EPHA2 and membrane bound EFNA1 engage in reciprocal signaling at cell-cell contacts and are important for maintaining adherens junctions [4,16,17,37]. In transformed cells overexpressing EPHA2, the receptor destabilizes adherens junctions and is mislocalized to membrane ruffles and to distinct punctae on the surface of the cell [4,16,17,37]. Delocalized EPHA2 is thought to signal in a ligand-independent fashion and lead to transformation. However, the possible contribution of soluble EFNA1 to this process has not been examined. To investigate this question, we first examined the cellular localization of EPHA2 in control and EFNA1 knockdown cells. In contrast to control-transfected cells, where EPHA2 had a more punctuate appearance on the cell surface and localized to membrane ruffles (Figure 5A,B), EPHA2 was primarily localized to cell-cell junctions and the peripheral membrane in EFNA1 knockdown cells (Figure 5C, D). EFNA1 localization in control cells also showed a punctate appearance and co-localized with EPHA2 on the cell surface and membrane ruffles. This pattern was reduced in shRNA treated cells confirming that localization was specific for EFNA1 and not the cross reacting proteins. We also examined the localization of EPHA2 in cells overexpressing either full-length EFNA1 or truncated EFNA1. In cells expressing full-length EFNA1 (Figure 6A, B), EPHA2 was enriched at cell-cell contacts and the peripheral membrane. In contrast, in cells overexpressing truncated EFNA1, EPHA2 and EFNA1 localization was more diffuse and was not enriched at cell-cell contacts (Figure 6C, D). This shows that EFNA1 is important for EPHA2 localization in these transformed cells, and suggests a mechanism whereby release of EFNA1 from the cell surface participates in the relocalization of EPHA2 during oncogenesis in some cell types.

Collectively, our results show that soluble EFNA1 is required for EPHA2-mediated oncogenesis in HeLa cells and affects the relocalization of EPHA2 from sites of cell-cell contact which occurs during EPHA2 mediated transformation.

## Discussion

It has been known since the early 90's that many cancer cell lines shed EFNA1 from their cell surface [21]. However, the significance of this has been unclear. Although this early work showed that EFNA1 from the conditioned media of cell lines was able to activate EphA kinase activity, subsequent contradictory work showing the apparent inability of soluble monomeric ephrin to activate Eph receptors led to the widespread generalization in the literature that ephrins must be membrane bound to signal [1,34,38-40]. The recent confirmation that soluble monomeric EFNA1 can activate EPHA2 suggests that soluble EFNA1 has the ability to signal beyond cell-cell contacts [31]. Indeed, our results show that soluble EFNA1 is important for transformation, and is a positive growth signal in HeLa and SK-BR3 cells. This provides evidence that the production the production of soluble EFNA1, at least some cancer cells lines, has physiological relevance.

A second controversial aspect of EFNA1 signaling in cancer cells is whether the protein promotes or inhibits oncogenesis. Our work provides some insight into the seemingly disparate ability of the same protein to both positively and negatively regulate oncogenesis. Within a single cell type we have found EFNA1 can promote or inhibit cell growth depending on whether it is presented in the conditioned media or membrane attached, and on expression levels. Specifically, overexpression of full length EFNA1 inhibited anchorage independent growth but had no effect on growth in 2D. Overexpression of soluble EFNA1 had little effect on growth in 3D but promoted growth in 2D. When soluble EFNA1 was provided in the conditioned media it had a positive role in the growth of cells in both 2D and 3D. This result indicates that the amount and whether it is presented in its membrane attached or soluble form contribute to whether EFNA1 is inhibitory or pro-oncogenic. Overproduction may interfere with EPHA2 trafficking or block EPHA2 interaction with soluble EFNA, especially when EFNA1 is membrane attached. Collectively, our results suggest that producing moderate levels of soluble EFNA1 in these EPHA2-positive cells promotes growth and transformation. Further work is required to determine how applicable these results are to other cancer cells that express soluble EFNA1. However, our finding that soluble EFNA1 is required for the growth of both a cervical and breast cancer cell line suggests that the positive role of soluble EFNA1 in promoting the growth of cancer cells is not restricted to one cell line or cell type. Indeed the recent finding that EFNA1 is found in the serum of patients with hepatocellular carcinoma, suggests that soluble EFNA1 is also relevant for in situ cancer [20].

The localization of EPHA2 away from cell-cell contacts is correlated with its transformation properties [16,17]. Our results show that soluble EFNA1 is important for this relocalization in HeLa cells. In cells deficient for EFNA1 or over-expressing full length membrane bound EFNA1, EPHA2 returns to sites of cell-cell contact and is no longer transforming. Whereas in cells overexpressing soluble EFNA1, the localization of EPHA2 resembles that of wild-type HeLa cells, and is not enriched at cell-cell contacts. These results suggest that soluble EFNA1 is involved in the relocalization of EPHA2 during transformation. One possibility is that soluble EFNA1 competes for binding of other ephrins to EPHA2 at cell-cell contacts and thereby draws EPHA2 away from cell contacts. If membrane bound EFNA1 is important for anchoring EPHA2 at cell-cell contacts and soluble EFNA1 relocalizes EPHA2 away from these sites, what causes EPHA2 to return to cell-cell contacts in EFNA1 knockdown cells? We have found that HeLa cells also express ephrin A2 and A4 (data not shown). Therefore, EPHA2 localization may be restored through an interaction with these ephrins. However, further work is required to determine how soluble EFNA1 contributes to EPHA2 localization and signaling, and to test these possibilities.

Ephrins and Eph receptors have well established roles at cell-cell contacts that are necessary for such developmental processes as tissue compartmentalization, axon guidance, and angiogenesis. Our results show that in addition to these important roles in signaling between adjacent cells, EFNA1 also has the ability to signal at a distance through the production of a soluble non-membrane attached isoform. It will be interesting to determine whether signaling by soluble EFNA1 has any roles during normal development as well as to elucidate further its role in promotion of cancer.

## Conclusions

There have been contradictory findings with regard to whether EFNA1 is pro- or anti-oncogenic and whether soluble EFNA1 is functional. We have shown that within a single cancer cell line EFNA1 has both pro and anti-oncogenic properties and that this behavior is influenced by whether EFNA1 is membrane attached or not, and the amount of EFNA1 produced. Soluble EFNA1 is important for the growth of two cancer cell lines and contributes to the relocalization of EPHA2 away from cell-cell contacts which accompanies transformation. We conclude that the ability of cancer cells to release EFNA1 is an important step in EPHA2-mediated transformation in a subset of cancer cells. To our knowledge, this is the first study to show that endogenous soluble EFNA1 positively contributes to the growth of cancer cells and to demonstrate that the physiological effects of EFNA1 signaling are not limited to cell-cell contacts.

## Methods

### Cell culture, transfection and RNA interference

HeLa cells were maintained in Dulbecco's Modified Eagle's Medium with 10% fetal bovine serum (FBS), 5% CO_2 _at 37°C. SK-BR3 cells were maintained in McCoy's 5A modified media + 10% FBS, 5% CO_2 _at 37°C. For EFNA1 knockdowns, siRNA and shRNA were designed to three EFNA1 specific sequences: (1) ^P5'^UGAGGACUACACCAUACAU GU3' (human specific), (2) ^5'^GAAGGACACAGCUACUACUAC^3'^(mouse and human) and (3) ^p5 ^UCCACAGGAGAAGAGACUU^3'^(human). siRNA targeting of EFNA1 was achieved by purchasing duplex RNA molecules containing these sequences from Sigma (Saint Louis, MO). For shRNA experiments, the following cDNA oligos were generated: A1 shRNAF1 (5'-gtaccgtgaggactacaccatacatttcaagagaatgtatggtgtagtcctcattttttggaag-3'); A1shRNAR1 (5'-aattcttccaaaaaatgaggactacaccatacattctcttgaaatgtatggtgtagtcctcacg-3'); A1shRNAF2 (5'-gtaccgaaggacacagctactactttcaagagaagtagtagctgtgtccttcttttttggaag-3'); A1shRNAR2 (5'-aattcttccaaaaaagaaggacacagctactacttctcttgaaagtagtagctgtgtccttcg-3'). These oligos contain 19 bases of sense and anti-sense strands of human ephrin A1 separated by a loop sequence, and flanked at either end by restriction enzyme sites. A terminator sequence was added to the 3' end, in between the antisense sequence and the 3' restriction site. The above oligos were annealed (A1RNAiF1+ A1RNAiR1; A1RNAiF2+A1RNAiR2) and cloned into KpnI and EcoRI sites, downstream of an H1 promoter, which had been inserted into a pcDNA3 backbone in which the CMV promoter had been deleted. All constructs were confirmed by sequencing. HeLa cells were transiently transfected with either short hairpin RNA (shRNA) containing plasmids using Lipofectamine Reagent (Invitrogen) following the manufacturer's instructions or with siRNA duplexes using Hiperfect Reagent (Qiagen, Carlsbad CA) according to the manufacturer's instructions. For the generation of stable colonies, transfected cells were split (1:10) and G418 (400 μg/ml) resistant colonies were selected. For EPHA2 knockdowns, siRNAs targeting EPHA2 were purchased and transfected with Hiperfect transfection reagent as per the manufacturer's instructions (Qiagen, Carlsbad CA). As a control for the siRNA experiments either AllStar Negative siRNA AF546 (Qiagen, Carlsbad CA) or a scrambled duplex of the above EFNA1-1 sequence was employed. EFNA1-GPI was PCR amplified from full length human EFNA1 cDNA using the following primers: Forward-tcggatccatggagttcctctgggc; Reverse-tcgaattcaccgatgctatgtagaac. The product was gel purified, digested with BamHI and EcoRI and ligated into the BamHI/EcoRI sites in pcDNA3 using standard techniques. This creates a truncated EFNA1 which lacks the GPI anchor attachment sequence and is constitutively secreted.

### Western blots and immunofluorescence

Antibodies used for western blots and immunofluorescence were anti-EFNA1 (anti-mouse EFNA1 Sigma, clone E7150 and anti-human EFNA1 (Santa Cruz), anti-βactin (Sigma, clone A1978, Saint Louis MO), anti-EPHA2 (clone D7, Upstate, Lake Placid NY). The anti-mouse EFNA1 antibody was used to detect exogenous EFNA1 whereas the anti-human EFNA1 antibody detected the endogenous EFNA1. For western blots, HeLa or SK-BR3 cells were lysed in NP-40 lysis buffer (20 mM Tris pH 8.0, 137 mM NaCl, 10% glycerol, 1% nonidet-P40, 2 mM EDTA) with Protease Inhibitor Cocktail (P8465 Sigma, Saint Louis MO). Lysates were incubated on a gyrator for 20 minutes at 4°C and then centrifuged at 14000 rpm for 10 minutes at 4°C. The soluble fraction was separated by SDS-PAGE and transferred to nitrocellulose. The blot was blocked with Tris-buffered saline (TBS)-TWEEN20 (0.1%) with 5% skim milk powder and probed with primary antibody overnight at 4°C. Blots were washed three times five minutes with TBS-TWEEN (0.1%) followed by a one hour incubation with secondary antibody in TBS-TWEEN20 (0.1%). After three washes, blots were developed using ECL Plus detection system (GE Healthcare, Buckinghamshire UK) following manufacturer's instructions. Relative expression was determined using ImageJ (NIH) software densitometry analysis in comparison to β-actin levels.

Immunofluorescence localization was performed under non permeablilizing conditions on HeLa cells plated on 4 chamber Labtek Permanox slides (Nalgene NUNC, Naperville, IL). Forty-eight hours after transfection, cells were gently washed twice with phosphate buffered saline (PBS). Cells were fixed using 3% formaldehyde, 1% sucrose in PBS for 30 minutes. Cells were then washed three times with PBS and blocked for 1 hour at room temperature with 3% BSA in PBS. After two washes with PBS, coverslips were incubated with primary antibody for one hour. After six washes with PBS, coverslips were incubated with secondary antibody for one hour. Slides were then washed six times with PBS and twice with dH_2_O prior to counter staining with 1 μM Hoechst 33342 (Molecular Probes, Eugene OR) for 1 minute. Coverslips were washed with dH_2_0, and mounted using ProLong Antifade mounting media (Molecular Probes, Eugene OR).

### Semi-solid agar assays

Anchorage-independent colony formation was analyzed in semi-solid agarose assays. HeLa cells were mixed (3000 cells/24 well) with 500 μl of 0.25% (w/v) warmed agarose in DMEM with 10% FBS (complete media). This layer was plated onto a bottom layer of complete medium/agarose (0.5%). For rescue experiments, plates were incubated at 37°C, 5% CO_2 _and each day the media was replaced with either conditioned media (100 μl conditioned media + 100 μl of fresh DMEM 10% FBS) that was previously prepared from the same population or conditioned media (100 μl conditioned media + 100 μl of fresh DMEM 10% FBS) from HeLa cells overexpressing EFNA1-ve GPI (soluble EFNA1). The rescue with recombinant EFNA1 used EFNA1-Fc (EA1-Fc) and Fc at 1 μg/ml in complete media and was exchanged as above. Colonies were examined with Leica DM IRM inverted light microscope and analysed using OpenLab 5.0 Software. Wells were divided into a 12 position grid and the number colonies were counted at each position. Alternatively, the media was drawn off, and the agarose was heated at 65°C for 5 min to dissolve the agarose and the colonies were counted using a hemocytometer. For the conditioned media rescue, data represent the average from three independent experiments from two independent EFNA1 knockdown clones +/- the standard deviation. A one way ANOVA (F(3,44) = 51.70, p < .001) followed by a Bonferroni's multiple comparison post test was used to determine whether the observed differences in average number of colony coverage between groups were significant. For the A1-Fc rescue, data represent the average from four independent experiments on one clone +/- the standard deviation. A one way ANOVA (F(3,8) = 13.89, p < 0.0015) followed by a Bonferroni's multiple comparison posttest was used to determine whether the observed differences between A1-Fc and Fc alone treated cells were significant.

For the overexpression of EFNA1 experiment, HeLa cells were transfected with pcDNA3, full-length EFNA1, or soluble EFNA -ve GPI. The next day, the transfected cells were split (1:10) and G418 (400 μg/ml) resistant colonies were selected. After approximately 1 week of selection the colonies were pooled together by trypsinizing, counted and plated as above in semi-solid media. The data represent the average number of colonies from four independent transfections +/- the standard deviation. A one way ANOVA (F2,9) = 5.897, p < 0.023) followed by a Bonferroni's multiple comparison posttest was used to determine whether the observed differences between vector treated cells and EFNA1 overexpressing cells were significant.

### Cell growth assay

Zero, twenty-four, forty-eight, and seventy-two hours following transfection, relative cell number was assayed by crystal violet staining. Cells were washed once with PBS and fixed with 10% formalin for 10 minutes at room temperature. Cells were washed two times with dH_2_O and incubated with 0.1% crystal violet for 30 minutes at room temperature. To remove excess stain, cells were washed with dH_2_O. Crystal violet was extracted using 10% acetic acid. Extracts were diluted 1:4 and their absorbance was measured at 595 nm using a VictorV 1420 Multilabel plate reader. Data represents the average growth from three independent experiments averaged from two independent EFNA1 knockdown clones +/- the standard deviation. Error bars represent the standard deviation. A two way ANOVA (F(3,80) = 42.28, p < .001) followed by a Bonferroni post test was used to determine whether the observed differences at each time point were significant. In all cases, the growth of the knockdown cells was diminished by 72 hours in comparison to control or rescued cells (p < .001).

### Apoptosis assay

HeLa cells were transiently transfected as described above. At 24 hours post-transfection, cells were scraped and pelleted, washed once in PBS, and resuspended in 1× Annexin Binding buffer (FITC Annexin V Apoptosis Detection Kit, BD Pharmigen). Cells were stained for flow cytometry using Annexin V-FITC and Propidium Iodide according to manufacturer's instructions (BD Pharmigen) and sorted on a BD FACSCalibur system (BD Biosciences). As a positive control for apoptosis, cells were treated for four hours with camptothecin (4 μg/ml) and processed as above.

## Competing interests

The authors declare that they have no competing interests.

## Authors' contributions

SCA and AWH contributed equally to this work. SCA, AWH, NS, and HL carried out the EFNA1/EPHA2 knockdown studies. JB performed initial knockdown analysis and obtained preliminary observations. AC carried out the apoptosis analysis. PLH conceived of the study, participated in its design and coordination, and drafted the manuscript and performed the immunofluorescence analysis. All authors have read and approved the final manuscript.
